# Reply to “Letter to the editor: methodological considerations in the benchmarking of AI-based protein–aptamer complex prediction”

**DOI:** 10.1093/bib/bbag309

**Published:** 2026-06-14

**Authors:** Jiani Zhao, Kha Tram, Hongbin Yan, Yifeng Li

**Affiliations:** Department of Computer Science, Brock University, 1812 Sir Isaac Brock Way, St. Catharines L2S 3A1, Ontario, Canada; Cytodiagnostics Inc., 919 Fraser Dr Unit 11, Burlington L7L 4X8, Ontario, Canada; Department of Chemistry, Department of Biological Sciences, Brock University, 1812 Sir Isaac Brock Way, St. Catharines L2S 3A1, Ontario, Canada; Department of Computer Science, Brock University, 1812 Sir Isaac Brock Way, St. Catharines L2S 3A1, Ontario, Canada; Department of Biological Sciences, Brock University, 1812 Sir Isaac Brock Way, St. Catharines L2S 3A1, Ontario, Canada

**Keywords:** Boltz-2 version, independent benchmark, benchmarking, DNA secondary structure, binding free energy, negative control strategy

## Abstract

We thank MD Kazim Okan Dolu for their interest in our published work and for the opportunity to clarify several points regarding software versioning, benchmark independence, secondary-structure generation, binding-free-energy terminology, and negative-control design. We acknowledge that several descriptions in the original manuscript could be stated more precisely. These clarifications improve the transparency and reproducibility of the study, but do not affect the reported analyses or main conclusions.

## Re: Boltz-2 version and access date are internally inconsistent; the reported release postdates the stated access month

In our work reported in [1], the models were initially accessed and set up in May 2025. For Boltz-2 specifically, however, the local environment was later recreated during the study, and we did not notice the resulting inconsistency in wording. After re-checking the result creation times and the local software environment, we confirmed that the Boltz-2 outputs reported in the manuscript were generated beginning in August 2025 using Boltz-2 v2.2.0, corresponding to release commit ac1c858. Thus, the reported Boltz-2 version is consistent with the analyzed outputs. Applying the general phrase “accessed May 2025” to Boltz-2 was imprecise, and we thank the author for pointing this out.

## Re: the benchmark includes six Boltz-2 training-set complexes; framing it as fully independent may overstate comparative neutrality

The phrase “independent benchmark” should be qualified when referring to Boltz-2, because it could be read as applying equally to all evaluated models. The benchmark dataset was constructed based on the AlphaFold3 training cutoff and is independent with respect to AF3, Chai-1, and RF2NA. Boltz-2 is the exception because it has a later training cutoff; consequently, six of the 11 complexes fall within the Boltz-2 training set. A more precise description is therefore that the dataset is a benchmark constructed based on the AF3 training cutoff. This overlap was explicitly reported in the Methods section, and Boltz-2 was further analyzed using seen and unseen subsets in the Results. Accordingly, the overlap was considered in the interpretation of Boltz-2 performance. This clarification does not affect the dataset construction, reported analyses, or main conclusions of the study.

## Re: RNAFold-derived secondary structures were used for nine DNA aptamers in the 3dRNA/DNA evaluation; DNA-compatible folding tools represent a more appropriate alternative

We agree that RNAFold-generated secondary structures are not ideal for DNA aptamers, and that DNA-compatible folding or secondary-structure prediction methods would be more appropriate and may improve the performance of the template-based 3dRNA/DNA workflow. This limitation was already discussed in the Discussion section.

In the Methods section, the statement “Secondary structures were obtained using RNAFold” refers to the RNAFold option integrated into the official 3dRNA/DNA web-server workflow. This option was used to evaluate the 3dRNA/DNA approach in a standardized and reproducible manner within the official server setting. We agree that explicitly stating that RNAFold was used as an integrated option within the 3dRNA/DNA web server would make this point clearer for readers and avoid possible misunderstanding.

## Re: Molecular Mechanics Generalized Born Surface Area (MM/GBSA) estimates derived from Molecular Dynamics (MD) trajectories should not be described as ground-truth or experimental binding free energies

We acknowledge that the x-axis label “Ground truth binding free energy” and the corresponding description in the Fig. 1 caption in [1] could lead readers to interpret these values as experimentally measured binding free energies. A more precise description for the x-axis is “MM/GBSA-calculated binding free energy of GT structures.” For clarification and consistency, the y-axis can be described as “MM/GBSA-calculated binding free energy of predicted structures.” Explicitly adding “MM/GBSA-calculated” helps clarify throughout the manuscript that these binding free energy values are computational estimates rather than experimentally measured binding free energies. This clarification does not affect the calculated values or the corresponding analysis.

## Re: describing negative pairs as “genuine nonbinding relationships” is stronger than the evidence supports

We agree that the phrase “genuine nonbinding relationships” is not precise, and a more accurate description is “putative non-cognate negative-control pairs.” Our intention was not to claim that these mismatched protein–aptamer pairs had been experimentally validated as nonbinding pairs. Rather, the mismatched negative set was designed to construct non-cognate control pairs while reducing the likelihood of obvious cognate or homologous binding relationships.

Regarding nonspecific binding, a similar concern was raised during peer review, noting that mismatched protein–aptamer pairs alone cannot fully exclude nonspecific binding. In response to the reviewers’ suggestion, in the final version of the paper, we added an AF3 shuffled-sequence negative-control strategy and incorporated pocket occupancy analysis to quantitatively assess nonspecific surface adherence. This clarification does not affect the calculated values or the corresponding analysis.

**Key Points**

- Version 2.2.0 of Boltz-2, released in August 2025, was used in our original study.
- The benchmark dataset was constructed based on the ALphaFold3 training cutoff.
- RNAFold for DNA secondary structure prediction follows the official 3dRNA/DNA web-server workflow.
- MM/GBSA was used to calculate the binding free energy of ground-truth structures.
- Both protein–aptamer pair mismatching and sequence shuffling were used as separate negative control strategies.

## Data Availability

There are no new data associated with this article.
